# Reflections on Adapting an Early Language Programme to Languages Other than English While Maintaining Active Ingredients

**DOI:** 10.1111/1460-6984.70361

**Published:** 2026-11-09

**Authors:** Carolyn Letts, Sean Pert, Ewa Czaplewska, Elaine Ashton, Cristine Jack, Helen Stringer, Cristina McKean

**Affiliations:** ^1^ School of Education Communication & Language Sciences (Visiting Fellow) Newcastle University Newcastle upon Tyne UK; ^2^ Division of Psychology Communication and Human Neuroscience The University of Manchester Manchester UK; ^3^ Institute of Speech Therapy University of Gdansk Gdansk Poland; ^4^ School of Education Communication & Language Sciences Newcastle University Newcastle upon Tyne UK; ^5^ Department of Education The University of Oxford Oxford UK

**Keywords:** Adaptation, early language intervention, languages other than English

## Abstract

**Background:**

Building Early Sentences Therapy (BEST) increases 3‐ to 6‐ year‐old children's comprehension and production of simple sentence structures by promoting abstract representations of underlying predicate argument structures (PAS). It has been shown to be effective with monolingual English‐speaking children. Important active ingredients include joint action routines, modelling to support mapping of meanings to PAS, and systematic variation of nouns and verbs within sentence frames. Number of exposures to a PAS and variation between sentence content are carefully controlled to ensure an intensive dosage. We describe the adaptation of BEST to languages other than English (LOTE), preserving the sentence models and variation that support these ingredients.

**Aims:**

To adapt BEST for languages other than English, remaining faithful to its underlying theoretical principles and retaining its active ingredients. To create a methodology for adapting interventions and related assessment materials.

**Methods:**

Two languages were adapted, Polish and Sylheti. Sentence structures from the BEST assessment and intervention were translated morpheme‐by‐morpheme. Principles laid out in The New Reynell Developmental Scales *Multilingual Toolkit* were then used to examine how PAS is conveyed (for example through word order or through case endings supporting relatively free word order), and to ensure close adherence to the original dosage type and intensity. Vocabulary equivalence and cultural appropriateness were checked. Therapy materials were created corresponding to the previous English‐based ones, retaining active ingredients for effectiveness. The adapted versions of the BEST intervention were piloted with one Polish‐speaking and one Sylheti‐speaking child who each had language difficulties in their home language; the children were identified through teacher/parent concern, language questionnaires, and non‐English adaptations of sections of a commonly used language test.

**Outcomes:**

Initial assessments of the two children suggested intervention should focus on comprehension and production of PAS in simple sentences. This was confirmed through BEST assessment results, and it was then possible to use a language‐adapted BEST intervention programme for each child.

**Discussion:**

To maintain the active ingredients of this intervention, it was important to ensure that suitable analogues of the target structures were identified, and underlying thematic role mapping maintained. This made it possible to evaluate whether and how the active ingredients of the intervention would apply when using adapted materials, drawing on knowledge of the target language and any available language acquisition data. For adaptation of this kind, consideration should be given to the intervention goals, ingredients and dosage, ensuring that these do not become lost in translation. Further studies are needed on applying the intervention in two or more languages and where code switching is commonplace.

**Conclusions/Implications:**

Careful adaptation enables intervention in a variety of languages, especially when this is not focussed on surface phrase order and grammar.

**WHAT THIS PAPER ADDS:**

*What is already known about this subject*

- The BEST intervention has been evaluated with English‐speaking children. However, no adaptation to date has been made for languages other than English.

*What this study adds to the existing knowledge*

- To our knowledge that is the first attempt to adapt an early language programme to languages other than English. The study provides principles that enable preservation of the principles and active ingredients of the programme.

*What are the clinical implications of this study?*

- The study provides a methodology for adapting interventions to different languages. It explores the resource impact of doing this while indicating why it is important nevertheless to do so.

## 1 Introduction

### 1.1 Why Adapt an Intervention to Other Languages?

When planning to use an intervention programme, practitioners are faced with a number of dilemmas when it comes to children who speak languages other than the one in which the programme is written. In many cases such children will be bi‐ or multilingual, navigating a majority language spoken in preschool (below age 5 in the UK) or school settings and one or more other languages spoken in the home. In the UK for example 20.4% of preschool and school‐aged children speak a first language other than English (DfE 2025), with over 300 different languages spoken by children attending British schools (Bailey and Marsden 2017). Just as in the monolingual population, some of these children will be at risk for generalised language difficulty that could lead to a diagnosis of developmental language disorder. In the UK, the Health and Care Professionals Council (HCPC: 2023), which regulates speech and language therapy practice, and the NHS England constitution (2023) both require equitable delivery of speech and language therapy services. This means that Speech and Language Therapists (SLTs) should be able to deliver assessment and interventions in children's home languages, support home language use, and work with interpreters when clients/service users/patients are bilingual. The UK professional body, the Royal College of Speech and Language Therapists (RCSLT) has guidelines for working with bilingual children (RCSLT, 2023) that further support this.

Lack of assessments and intervention materials in other languages (in this case other than English) can make these requirements difficult to meet and mean that children may go unidentified and/or fail to receive appropriate intervention. SLTs may have little experience of working with interpreters and so are unable to use their linguistic analysis and phonetic transcription skills optimally to ensure these principles are adhered to, and a proportion of SLTs have been shown to be unaware of recent guidelines on best practice (Sharpe and Perovic 2023). While it may be tempting to provide assessment and speech and language therapy intervention only in English, this will fail to take into consideration the child's skills in their home language and may serve to alienate them from members of their family and community who do not speak and use English routinely. Without opportunities to support the development of the child's home language, language attrition is likely to lead to monolingualism in the second language (Kohnert 2013). Furthermore, providing intervention in the child's home language has been shown to facilitate the development of their second language (see Nair et al. 2023 for a recent review).

### 1.2 Building Early Sentences Therapy (BEST)

BEST (McKean et al. 2025a) is a play‐based early language intervention for children between the ages of 3;0 and 6;0 years with significant language difficulties. It centres on targeting specific sentence frames and their associated thematic roles and focuses on language input, which means children can listen and join in at their own pace. While opportunities are provided for responses from the child, there is no expectation that they will respond orally unless they are comfortable doing so. BEST is usually delivered in a group context of two to six children, so the children can hear sentences on multiple occasions, however, it can also be delivered one‐to‐one. Content words and morphology are also signed to the children; therefore, the intervention is a multi‐modal approach. Signing is based on the signing systems *Makaton* (Walker and Armfield 1981) and *Paget Gorman Signed Speech* (Rowe 1981). Use of sign supports children in mapping spoken content words to their meaning, and hence the overall predicate‐argument structure of the sentence. Signing of morphology highlights the morphological frame within which content words occur. Furthermore, signing is thought to reduce cognitive processing load for the children. This is discussed in Trebacz et al. (2024) which provides evidence supporting improved efficacy when signing is incorporated into the intervention.

Children eligible and likely to benefit from BEST are identified through the *BEST Assessment*, which samples the structures covered by BEST and captures the child's expressive language ability when unsupported. The assessment is also used to measure changes over time. A picture book format is used, and children are asked to tell the examiner what is happening in each picture.

BEST intervention sessions are scripted, and each session follows the same structure. Children hear the target structures multiple times with accompanying signs while watching toys being manipulated. They then have the opportunity to produce the structures. Homework activities are provided for parents after each session, reflecting the activities that were carried out in the session. A total of 16 scripted intervention sessions are provided, with assessments after session eight and session twelve. Depending on the outcome of these assessments, the intervention is either continued or terminated if sufficient progress has been made.

Full Details of BEST, Together With the Assessment and Intervention Materials Can be Found at: https://Research.ncl.ac.uk/best/

BEST has been delivered successfully in several clinical contexts in the UK. A quasi‐experimental study in 2024 provided evidence that BEST is more effective than ‘treatment as usual’ for expressive language in preschool children with low language ability, while also supporting the use of signing to accompany spoken input (Trebacz et al. 2024). A cluster randomised control trial comparing BEST with an Adapted Derbyshire Language Scheme (based on Knowles and Masidlover 1982), found gains in comprehension and a similar advantage for expressive language. Furthermore, there was continued significant progress for the BEST group after intervention, suggesting the intervention provided a boost to accelerate language learning (McKean et al. 2025b). The focus on sentence structure suggests that BEST lends itself to be adapted for use with children who speak languages other than English. However, to our knowledge, there are presently no published frameworks available for adapting interventions to different languages.

### 1.3 Theoretical Drivers and Active Ingredients of BEST

BEST is based on usage based/constructionist theories of language acquisition as outlined in Tomasello (2003). Acquisition is viewed as depending on two intrinsic and innate psychological/learning processes, intention reading and pattern finding. In the first case, the child assumes language from others is motivated by communicative intent and that the interlocutor is attempting to impart meaning, be it to pass on information, ask questions or issue instructions. The child's task is to work out what this meaning is and consequently, in BEST, input is carefully structured to facilitate this. In the second case, pattern finding, the child is motivated to note recurring patterns in the language; this in turn will give them the ability to understand and use language in an increasingly broad range of situations. In BEST, this is facilitated through frequent exposure to the same sentence structure while systematically varying its components in a way that promotes awareness of the underlying pattern rather than of a particular sequence of words. The development of these abstract representations then supports generalisation after the intervention is completed.

These principles underpin the active ingredients of BEST, which are listed below, together with implications for languages other than English (LOTE) adaptations. In considering such adaptations, guidance is followed from the *New Reynell Developmental Language Scales Multilingual Toolkit* (Letts and Sinka 2011). While this guidance was developed to inform test adaptation, its underlying principles can equally apply to intervention. The guidance suggests first establishing the linguistic focus of test items, or in this case the intervention materials, followed by reflection on how this focus is manifest in the target language. This leads to a set of questions to consider, moving on to constructing the adaptation.

Taking each active ingredient in turn, implications for adaptation to a LOTE would be:

1. *Joint action routine to scaffold child's ability to infer communicative intention*:
  This involves, firstly, creating a joint attentional frame between child and adult through a play routine. The link between a descriptive sentence and an event within the routine is then made explicit allowing the child to make inferences about meaning. When making adaptations, care is needed to ensure that the routines used are not culturally alien to the child. However, where the child has regular exposure to a preschool education environment, they are likely to have experience of similar interactions and routines with adults.
2. *Role reversal established Through routine*:
  Here the child is given the opportunity initially to imitate and then to experiment with directing communicative intention to the adult. Although not an issue for the languages/cultural situations piloted here, it is possible that there may be cultural constraints on how children interact with adults and appropriate adjustments would need to be made, such as directing utterances at another child or a puppet character (see for example Burdelski 2010, describing use of strategies to promote polite language towards adults in a Japanese preschool).
3. *Schematisation/categorisation promoted by quantity and distribution of types/tokens*:
  To move the child towards an abstract representation of a simple sentence structure, BEST exposes the child to sentences where one element is varied while the rest of the sentence remains the same. For example, the grammatical subject (teddy) of ‘teddy is jumping’ is varied by using sentences like ‘the girl is jumping’, ‘the boy is jumping’ and so forth. Similar sets of stimuli are constructed for different ‘slots’ in the sentence frame, for example the verb or the grammatical object in a Subject‐Verb‐Object (SVO) sentence. For adaptations, care must be taken that the sentence frames and the items that can be varied within them work for the target language. Basic word order differs across languages and may be relatively free, with thematic roles such as ‘entity carrying out the action’ or ‘entity affected by the action’ being signalled by case inflections rather than word order. Word order factors were important for the languages considered here. Simple sentence word order is different in Sylheti than English, and Polish has a rather freer word order than English. In some languages it may also be acceptable to omit parts of the sentence where these are obvious from the context, something which is not permissible in English.
4. *Repeated exposure to sentence construction pairs that have the same PAS*:
  This facilitates the formation of abstract representation of structures, due to repeated exposure. It should be noted that a straight translation may result in a different PAS for one or more of the items in a set. For example, in the Polish adaptation described in this paper, the Polish equivalent of the sentence ‘X is riding a bike’ in the SVO structure would be reserved for situations where the emphasis is on the means of transportation one uses to reach a destination (i.e. ‘bike’ versus ‘car’ or ‘on foot’ and so forth). Where a straightforward description of a physical activity is intended, the appropriate Polish sentence would equate to ‘X is riding on a bike’, a Subject‐Verb‐Adverbial (SVA) structure involving use of a prepositional phrase. This rendered BEST structures that featured ‘*riding’* as a verb unsuitable for the adapted version of BEST and so alternative stimuli were found in these cases.
5. *Non‐overlapping sets (e.g. patients never agents and vice versa)*:
  Using different sets of nouns in each sentence ‘slot’, with no nouns appearing in more than one slot, is thought to facilitate making analogies around sentence structure (McKean et al. 2025b). While this is straightforward to implement in different languages, it should be born in mind that in highly inflected languages such as Polish that feature case morphemes, the same noun will have a different form when e.g. subject versus object. Moving on from the BEST sentences, the child may need additional support to recognise that these different forms are actually variations of the same lexical item.
6. *Use of toys supports identification of PAS roles*:
  Play is constructed around familiar toys, with the meaning of each sentence acted out and illustrated using the toys. It is important to ensure that toys are culturally appropriate for the child and to make substitutions if necessary.
7. *Rotation Through different constructions*:
  Structures are revisited in subsequent sessions to ensure retention.
8. *Visual Referents to support, including use of sign*:
  In addition to toys, visual support is provided by use of signs accompanying the input sentences. In the English language version of BEST, Makaton signs (https://www.makaton.org/) are used for content words and Paget‐Gorman signs (Rowe 1981) for function words/inflections. Through hearing inflections and function words, together with visual signs, the child is made aware of them and is facilitated to use them in the target structures. This is relatively straightforward in English, but other languages may have more complex systems of e.g. inflections, where use of multiple different signs may not be so helpful. It was necessary to consider this when using signs with the Polish version of BEST.
9. *Exposure over multiple sessions with multiple opportunities to use target construction expressively*:
  Together with revisiting structures covered in earlier sessions, this aids retention. Here homework activities in Polish and Sylheti further supported this in line with the English version of BEST.

Our aims were as follows:

1. To adapt BEST for languages other than English, remaining faithful to its underlying theoretical principles and retaining its active ingredients.
2. To create a methodology/framework for adapting interventions and related assessment materials. To our knowledge, this has not been attempted before, and there is a dearth of information on how to make such adaptations.

## 2 Method

Consent for participation was obtained from the children's parents and from participating schools.

### 2.1 Structures Targeted by BEST

The structures targeted in the English version of BEST, including increasingly complex PAS and accompanying function words and inflections, are summarised in Table 1. Comparable structures in the target languages were then identified.

**TABLE 1:** Structures targeted in English BEST.

Predicate‐argument structure PAS)	Sentence structure	Example
Verb plus one argument	SV	*The lady is jumping*
Verb plus two arguments	SVO	*Teddy is eating a banana*
Verb plus three arguments	SVOA	*The baby is pouring milk into a cup*

Key: S = Subject; V = Verb; O = Object; A = Adverbial.

### 2.2 Target Languages

In order to decide what languages to focus on, a first step was to gain information from participant schools regarding children's home languages. Schools were all in the North East of England. Three languages spoken at home by large numbers of children were identified: Polish, Sylheti and Mirpuri. Consequently, adaptations were made of all the materials needed to identify prospective children for the BEST intervention, and of the intervention materials themselves, for these languages. Two children were eventually recruited to the study, one Sylheti and one Polish speaker, so these two languages were then focused on.

Polish and Sylheti are spoken by substantial minority populations in the UK and children recruited for BEST from these communities are likely to be bilingual, or emerging bilingual, speakers. When these children start preschool education (at age 3 or 4 years), they will typically have been exposed more to the home language and may have only limited exposure to English. An initial consideration when adapting the intervention, was whether the structures facilitated through BEST actually have relevance in the target language. We can however say with some confidence that the PAS structures that are focused on in BEST will be important regardless of language, as all children appear to make the transition from single words to simple multiword sentences between about 18 months and two years. Furthermore, there is evidence that children become aware of word order constraints that operate for their language(s) from the beginning of this period (Zhu et al. 2022; Montanari 2009). In contrast, passive constructions, not included in the BEST intervention and which develop much later, do not exist in some languages (Sierwierska 2013), and in many others are used infrequently. To take another example, systems of grammatical inflections vary considerably across languages in terms of complexity, frequency of use and transparency and this will be reflected in age of acquisition (and again some languages have no or very few grammatical inflections; Letts 2024). Given BEST's focus on sentence structure however, it was safe to assume that intervention based on BEST principles should be applicable to any language.

### 2.3 Language Characteristics

We were aware at the outset of some features of these languages which could impact our adaptations:

1. Sylheti does not make extensive use of inflections but does have a different standard word order from English (see point 3 in the list of active ingredients in the Introduction above). For the sentences used in BEST, a subject‐object‐verb (SOV) word order applies. While this would clearly be reflected in the translations, it should not have any impact on processing load, as it would just be a re‐ordering of the sentence elements.
2. Polish is a highly inflected language with case endings on nouns as well as agreement inflections on verbs. Furthermore, nouns have a grammatical gender which affects the form the inflections take. While BEST has a primary focus on sentence structure, in the English version, inflections and function words are consistent across sets of sentences and form part of the sentence frame. This is not the case with Polish. It was also unknown at that stage whether PAS is mediated extensively by case endings, or whether the increased functional load involved in processing these would impact the children's response to the intervention. (Again, see point 3 in the list of active ingredients in the Introduction above.)

As the English version of BEST was the starting point here, we tried to ensure a faithful translation of the English stimuli. However, it was also important to consider any relevant developmental sequences. As already noted, acquiring word order for PAS appears to be relatively consistent across languages in terms of when children begin to produce simple sentences and be sensitive to word order. Acquisition of inflections however is likely to be variable and will depend on features such as frequency, consistency of form, transparency regarding the contexts in which they appear, and phonological salience and ease of production (Letts 2024). Work on acquisition of inflections has been largely limited to English plus a small number of other languages, mainly European (see for example Leonard and Kuesner 2019). Unfortunately to our knowledge there is very little relevant work on Polish.

*Approach to devising adapted BEST stimuli*:

The starting point for adapting items was a morpheme‐by‐morpheme translation of the target sentences following the translation protocol devised by Pert and Stow (2003). This ensured that no key elements in the target sentences were omitted. To ensure that the active ingredients listed in the Introduction were not compromised, reference was made to the NRDLS *Multilingual Toolkit* (MLT: Letts and Sinka 2011) sections on ‘Selecting/Naming Objects’, ‘Sentence Building’ and ‘Verb Morphology’. For example, a number of issues are described in the MLT which may render finding suitable vocabulary problematic, including cultural acceptability and familiarity to young children, whether a single word in one language is expressed through a phrase in the other language, and whether there is similarity in meaning where words sound similar or have been ‘borrowed’ from one language to the other, as with cognates. Care was taken to avoid such problems as far as possible, and where alternatives were available, to choose those most widely used within the child's community.

Sentences were each placed into a table or ‘grid’, with each morpheme in a single cell. This made it possible to establish the function of each morpheme and note differences from the English structures.

### 2.4 Sylheti

Translations were provided by a native speaker of Sylheti and experienced bilingual Speech & Language Therapy Assistant based in the UK, together with the second author who has extensive experience working as an SLT with Pakistani Heritage speakers. Table 2 shows the same constructions from the BEST assessment as given above, for Sylheti:

**TABLE 2:** Examples of Sylheti adapted sentences.

English stimulus	*The lady is jumping*					
Morpheme by morpheme translation	beti	fald	er			
English	lady	jump	*pres prog (is)*			
**Stimulus**	***Teddy is washing a banana***					
M by M Translation	teddy	kola	dho	ar		
English	Teddy	banana	wash	*pres prog (is)*		
**Stimulus**	***The baby is pouring the milk into a cup***					
M by M Translation	bacha	dood	khafor	bithre	dal	air
English	baby	milk	cup	inside	pour	*pres prog (is)*

Key: *pres prog =* present progressive inflection.

There are no articles in Sylheti (i.e. *a* and *the* in English). There are no case inflections, and word order is not flexible. However, Sylheti follows a SOV or SOAV word order, and prepositions (e.g. ‘inside’) are placed after the relevant noun. These sentences all have progressive aspect, with this shown through an inflection on the verb rather than an auxiliary verb plus main verb inflected with *ing*, e.g. ‘is pouring’, as happens in English.

### 2.5 Polish

Translations were provided by the 3rd author, a native speaker of Polish based in Poland and a professor of psycholinguistics with a background in psychology and speech and language therapy. She continued to advise on the adaptations going forward. Table 3 gives examples of Polish adapted sentences, taken from the BEST Assessment:

**TABLE 3:** Examples of Polish adapted sentences.

English Stimulus	*The lady is jumping*								
Morpheme by morpheme translation	pan	i	skacz	e					
English	lady	*nom*	jumps	*agr*					
**Stimulus**	***Teddy is eating a banana***								
M by M	miś	Ø	je	e	banan	a			
English	teddy	*nom*	eats	*agr*	apple	*acc*			
**Stimulus**	***The baby is pouring milk into a cup***								
M by M	dzieck	o	wlewa/nalewa	Ø	mlek	o	do	kubk	a
English	baby	*nom*	pours	*agr*	milk	*acc*	into	cup	*loc*

Key: *nom =* nominative case; *acc =* accusative case; *loc =* locative case; *agr =* agreement (of verb with subject of sentence).

There are several features of Polish that become apparent when following this process. Firstly, like Sylheti, there are no articles in Polish. There is no present tense verb form that conforms to progressive aspect and instead a simple form is used, with an inflection for agreement with the subject of the sentence. Secondly, nouns are inflected for case, which means that PAS can be deduced from noun inflections, regardless of word order. Default word order in Polish is SVO however and this is adhered to in the translated sentences. In the examples given above, *nominative* inflections indicate the subject/agent of the sentence, *accusative* indicates the object/patient, and *locative* indicates adverbial/locative. Note that in some instances, the inflection slots for agreement and case are marked as zero (Ø): in these specific verbs and nouns there is no inflection. Agreement rules depend on specific verb conjugations, while case inflections are affected by grammatical gender of nouns.

### 2.6 Language Specific Issues Encountered While Making Adaptations

Adaptation of BEST to Sylheti was relatively straightforward; once word order was changed to reflect the SOV language, BEST sentences in English mapped straightforwardly on to equivalent Sylheti structures. For the purposes of the BEST intervention and assessment, there were no issues arising in terms of vocabulary. The present progressive verb inflections used throughout varied according to the individual verb (*er, ar, air*), although these are phonetically similar to each other. This could mean, potentially, that the sentence frames would be less consistent to the child than the English version. However, it should be borne in mind that there is no research to our knowledge on the acquisition of Sylheti and in particular, no Sylheti version of McArthur‐Bates Communicative Inventories (see https://mb‐cdi.stanford.edu/about.html) as was available for Polish.

For Polish a number of issues arose:

1. The phonological structure of Polish words is more complex than English, with multiple consonant clusters. Arguably phonological structure could affect rate of acquisition of vocabulary (Schwartz and Leonard 1982; Bleses et al. 2008). There is also evidence that exposure to complex word initial clusters in Polish accelerates production of such clusters in Polish speaking children as compared to English speaking children (Tamburelli et al. 2015). To be sure that the vocabulary used in BEST was appropriate, it was checked against the Polish version of the McArthur‐Bates Communicative Inventories (MB‐CDIs) (Smoczyńska et al. 2015), which provides a checklist of early vocabulary in Polish speaking children, and a frequency list provided by Zgółkowa (2016). We were able to ascertain that children falling within the age‐range targeted by BEST should be familiar with this vocabulary.
2. In common with other Slavic languages, nouns frequently have a ‘diminutive’ form which is used when talking to small children. Although a decision was made to in principle use diminutive forms where these were likely to be more familiar to the child, in fact this did not arise with any of the vocabulary items used in BEST.
3. SVO English expressed through SVA in Polish, in this case regarding the verb *ride*: In Polish it is not possible to talk of ‘riding X (e.g. horse)’, and a structure corresponding to ‘riding *on X*’ is used. Substitutions were made to avoid this, for example in intervention session 2, set D, *brushing* is substituted for *riding*.
4. Signing inflections: given the complexity of Polish inflections and the presence of multiple forms, a decision was made to just use one, neutral, sign to indicate a suffix.

The BEST assessment, together with adapted sections of the Comprehension and Production Scales from the *New Reynell Developmental Language Scales* (NRDLS: Edwards et al. 2011, see below) were piloted on a Polish/English speaking child aged 6:1 and a Sylheti/English speaking child aged 6;3, both typically developing, and living in the UK. These children would be expected to be able to respond appropriately to all the stimuli in the assessment, and the purpose was to check for any practical issues that might come up and for any vocabulary that might be problematic. For the latter, it was possible that, for the bilingual children who were the focus of study, the influence of English may have brought about changes in their LOTE language vocabulary use. No issues arose with the BEST assessment. However, there were some issues with the adapted sections of NRDLS. For Sylheti, no equivalent word could be found for *wave* (as in greeting or saying goodbye), as the gesture is not used in the culture of Sylheti speakers. The same is the case for speakers of Mirpuri and Punjabi. In Polish, the word *auto* for ‘car’ was substituted for *samochód* following piloting, and *klapie* recommended for ‘clapping’, as being the forms most commonly heard and used by Polish‐speakers in the UK.

### 2.7 Piloting: Single Case Studies

Children for the intervention pilot studies were identified by teachers as potentially being able to benefit from BEST intervention in their home language. Questionnaires for parent/caregivers (conducted as semi‐structured interviews) and teachers were used to evaluate the child's use of and exposure to the home language and English. Language assessments were carried out in both the home language and English. It was important to identify children who were experiencing difficulty in both their languages, rather than poor performance in one of their languages resulting from limited exposure. The language assessments consisted of adapted sections of the NRDLS, taking into account overall score and performance specifically on section C (verbs) and D (sentence building), these being the sections most relevant to the structures targeted in BEST. The BEST assessment was also carried out. Home language assessments were carried out working with an interpreter.

Assessment led to one Sylheti‐speaking child aged 3;10 years and one Polish speaking child aged 4;07 years, being identified and recruited to participate. In each of these cases the child performed poorly in both their languages. A further Polish‐speaking child was assessed but did not meet eligibility criteria as their knowledge of Polish appeared to be very limited in comparison with English. This was confirmed by carrying out the full standardised version of NRDLS in English, with the child scoring just within the age‐range expected for their age. The BEST programme in respective home languages was then delivered to the two children identified, through twice‐weekly sessions over 12 sessions for Sylheti and over 14 sessions for Polish. Sessions lasted for about 30 minutes and were carried out in school, in a quiet room away from the classroom. Originally 16 sessions were scheduled for each child, but absences meant that these could not all be carried out. An English‐speaking SLT (4^th^ author) and an interpreter worked together to deliver the intervention, with the SLT signing words spoken by the interpreter.

Unfortunately, due to unforeseen difficulties with intervention delivery and with working with interpreters we were unable to collect data which allows for the efficacy of the intervention to be evaluated. These difficulties arose from the sociolinguistic characteristics of the context and in particular the children not being used to speaking their home language in the school environment. However, to maximise the learning from this study we have prepared two publications—one which describes the process of adaptation (this paper) and one which describes the learning with respect to providing intervention in home language via interpreters (Ashton et al. unpublished manuscript). So we do not have evidence that the adaptation worked but at this point we want to share it as a principled way to consider active ingredients cross‐linguistically. For further information on the pilot study, please see the protocol provided as supplementary information.

## 3 Discussion

The aims for this study were to adapt an intervention, BEST, to two languages and at the same time establish a methodology for making such adaptations. It was necessary to remain mindful of the underlying principles of BEST and its active ingredients.

### 3.1 Adaptation of BEST: Maintaining Active Ingredients

Key linguistic components of BEST are repeated exposure to pairs of sentences with the same syntactic frame and the ‘swapping out’ of sentence elements with each other while maintaining the same sentence frame. In both Polish and Sylheti, it was possible to make a fairly direct translation from English of the simple sentences in BEST, making changes to word order as appropriate for Sylheti, and arrive at a comparable set of sentence frames. When working on simple PAS in sentences, it would seem that making a basic adaptation from one language to another is straightforward, provided care is taken to be mindful of how PAS works for individual structures in the target language, such as the ‘ride on a X’ example given above for Polish.

Where sentence structure word order is relatively fixed, as in English and Sylheti, we can be confident that, having acquired simple sentence structure as a result of the structured exposure BEST provides, the child can then carry this over to other similarly structured sentences they hear in the language and so continue to make progress. This may be less likely where word order is more variable with cues for PAS provided by other means such as case inflections. While as with Polish there is likely to be a default word order, the child is nonetheless exposed to only a subset of the possible sentences they will hear with limited carryover to differently ordered sentences. Here, an approach which explicitly contrasts sentences with different word orders, but consistent case endings, may also be useful.

Sentences in BEST are grouped to reflect identical frames within which it possible to vary components. In the English version, all the sentences contain third person singular present progressive verbs of the *is Xing* form for example, as in ‘the teddy is walking’. This may be less consistent in languages where different conjugations lead to different verb inflections, or where case inflections are dependent on features such as gender and number, or indeed where inflectional systems operate quite differently from English. Examples of the latter are root‐pattern morphology co‐existing with inflections in Arabic languages (Ravid and Farah 2009), or the system of mutations in Welsh (Thomas and Gathercole 2007). Again, explicitly contrasting verb conjugation‐ or inflection sets may be necessary.

The approach used to make these adaptations took the English version of BEST as a starting point and worked from morpheme‐by morpheme translations from English. This has the advantage that materials and scripts already developed for the English version could be used, albeit with some minor modifications being required. Furthermore, the English intervention is tried and tested, with demonstrated efficacy (McKean et al. 2025b). However, this was facilitated by relative similarities between the structure of these languages and English permitting us to make adaptations that retain the active ingredients of BEST effectively. The Sylheti adaptation results in a consistent set of structures where components can be interchanged with little disruption. With Polish however, consideration needed to be given to factors such as use and function of case endings, transparency and consistency of inflections on verbs and nouns (including case endings), and the degree to which a default word order reflects the language that the child hears predominantly in their everyday environment.

Direct adaptation from one language to another may be difficult where the languages differ substantially from each other. In this case, a better approach may be to start from identifying suitable structures from scratch in the target language, following BEST principles, and develop appropriate materials around these. This might also be a better way forward where major modifications to vocabulary used in BEST would otherwise be needed to ensure familiarity to the child. Where the highly structured interaction that underpins BEST is culturally inappropriate, the principles underpinning BEST could still be relevant in developing target structures for a different style of intervention.

### 3.2 Establishing a Methodology for Adaptation

If the decision is made to make a direct adaptation from another language, we would recommend the morpheme‐by morpheme approach described here as an initial starting point, using a linguistically aware expert native speaker of the target language to provide translation and information about the language structure. This method may also give an indication whether an adaptation or a ‘start from scratch’ approach is better, as the typography of the target language becomes clear.

With the BEST intervention and assessment and the two languages explored here, there were no real issues with vocabulary; direct translations of the English vocabulary could be used, and the items were familiar to children in the 4–6‐year age range. However, some issues with vocabulary were found when piloting the adapted sections of NRDLS. Principles around adapting vocabulary in assessments are laid out usefully by Dale (2015), as well as in the NRDLS *Multilingual Toolkit* (Letts and Sinka 2011).

### 3.3 Bilingual or Monolingual Intervention?

In this study, intervention was delivered to bilingual or emerging bilingual children in the UK context, exclusively in the child's home language. Any adaptation for use with children in countries where the target language is dominant, may have turned out somewhat differently. Communities of speakers within different countries may use vocabulary influenced by other languages spoken around them (see the ‘car’ example for Polish) and may start to diverge from that of the ‘home’ country. This should be taken into account when making any adaptation.

A further consideration is the degree of carry‐over from intervention in one language to another in bi‐ or multilingual contexts. Here the focus and rationale of the intervention is important. If the intervention is aimed at helping the child develop strategies for learning new vocabulary for example, it is likely that the strategies will apply regardless of language. For the sentence structures targeted in BEST, this will depend on how closely the structures match. With the difference in underlying word order between English and Sylheti, carryover is less likely to happen. Further research could explore an intervention which contrasted the two different word orders in a structured way while applying the underlying principles of BEST.

Arguably for children living in a bilingual environment, intervention should be delivered in both languages. This raises questions around whether the interventions should be kept separate, for example providing intervention in school in the majority language complemented by intervention in the home in the home language. A major problem encountered with our pilot studies was the artificiality of trying to create a home language environment in a predominantly English‐speaking school setting and the children's subsequent reluctance to engage. In a different cultural setting where languages are used interchangeably and where code‐switching is likely to be commonplace, different decisions would be required around how to accommodate both languages, together with code‐switching, in the intervention (see Pert and Letts 2006 for more information on code‐switching within a minority community in the UK).

### 3.4 Challenges to Adaptation and Limitations to this Study

The process of adapting the intervention, going through morpheme‐by‐morpheme translation followed by consideration of whether the structures work in the target language and then checks for appropriacy of vocabulary, is time consuming and requires collaboration between the SLT and expert linguistically aware native speakers of the language. The SLT can provide guidance on ensuring the intervention does not lose focus on the active ingredients, and the adult language informant brings both cultural knowledge and knowledge regarding the acceptability and appropriateness of any proposed alterations from the original version. Furthermore, lack of research into acquisition in the target language, for both mono‐ and bilingual (or indeed multilingual) children can result in some decisions seeming somewhat arbitrary. For example, lack of information on the acquisition of Polish inflections led to a ‘place holding’ generic sign being used when signing the BEST sentences.

A major limitation to this study was that we were unable to gain data that allowed evaluation of the effectiveness of the adapted versions of BEST. Difficulties arose around the artificiality of the intervention environment which entailed use of the home language in a setting where this would not normally occur, and with the complex coordination of input from the SLT and interpreter. These issues are explored further in Ashton et al. (in preparation) . Clearly more research into delivery of intervention with bilingual children is needed; an account of adaptation of an intervention such as described here should feed into such research.

## 4 Conclusion and Recommendations

This paper sets out recommendations for the adaptation of BEST and similar interventions into languages other than English. These can be summarised as follows:

1. Consider whether the highly structured input that characterises BEST is culturally appropriate.
2. Morpheme by morpheme translation is recommended initially to establish how PAS and inflections work in the target language.
3. Consider whether word order of sentence components (S, V, O etc.) is the same in both languages, or differs.
4. Consider whether word order is relatively free and if so whether there is a ‘default’ order that can be followed in BEST.
5. Be aware that vocabulary may not map exactly from one language to another, or that PAS for specific verbs may work differently (as with ‘riding’ in Polish).
6. Inflectional morphology varies considerably between languages and in terms of age of acquisition. Consider whether it is appropriate to include this as a focus of the intervention.

A substantial investment of time is required, along with clear knowledge of the active ingredients, access to expert speakers of the target language and knowledge of its culture. However, in areas where there are substantial populations speaking the target language, this is an investment that is likely to be cost effective in the long term. It is recommended that detailed records of the process are kept so that changes can be made if indicated following implementation. In clinical practice, a single case study, service evaluation or quality improvement process would contribute to the gathering of evidence at a local level and facilitate sharing evidence on a national scale.

Further research is also needed, exploring adaptations to a wider range of languages. The adaptations considered here were made assuming a monolingual intervention which in many circumstances may not be appropriate. Further work is also needed on using BEST to work in two (or more) languages with the child and on working in situations where code switching is common.

BEST Resources for Sylheti, Polish and Mirpuri Can be Found at: https://Research.ncl.ac.uk/Best/Bilingual‐best/

## Funding

This research was funded by the Heather van der Lely Foundation. We thank the foundation for this funding and for its flexible and supportive approach during the COVID pandemic. Funding for the development of Building Early Sentences Therapy (BEST) was provided by Newcastle University.

## Ethical approval

The project has ethical approval from Newcastle University Research Ethics Committee. Informed consent was obtained from parents of child participants and from headteachers of participating schools.

## Conflicts of Interest

The authors have no financial or other conflicts of interest in respect of this article.

## Supporting information




**Supporting information**: jlcd70361‐sup‐0001‐SuppMat.docx

## Data Availability

Resources developed for this study are available on the BEST website:  https://research.ncl.ac.uk/best/bilingual‐best/No profits are made by the authors.
