# Supplementary material for: Reflections on Adapting an Early Language Programme to Languages Other than English While Maintaining Active Ingredients
Source: Int J Lang Commun Disord. 2026 Nov 9;61(6):e70361. doi: 10.1111/1460-6984.70361 (PMC13749059; doi:10.1111/1460-6984.70361)
Supplement: Supplementary file 1 [file JLCD-61-0-s001.docx]

LIVELY Bilingual Case Series

Study Protocol

Cristina McKean – Sean Pert – Carolyn Letts – Ewa Czaplewska – Christine Jack – Elaine Ashton

| Step 1 | Time needed |
| --- | --- |
| Teacher briefing & set up expectations | 1 meeting per school  1 week |
| Step 2 |  |
| Teacher identifies candidate children | 2 weeks |
| Step 3 |  |
| Consent obtained from family for Assessment & Study | Interpreter setup 1 week (overlap above)  Meetings 2 weeks |
| Step 4 |  |
| Assessment | 3 sessions per child  3 weeks |
| Step 5 |  |
| Comparison to Eligibility Criteria | 1 weeks |
| Step 6 |  |
| Intervention delivery set up | 2 weeks (overlap with above) |
| Step 7 |  |
| Randomisation to baseline set | Simultaneous to Step 6 |
| Step 8 |  |
| Baseline assessments | Max 5 weeks |
| Step 9 |  |
| Intervention delivery & Assessments | 7 weeks |
| Step 10 |  |
| Outcome Assessment | Gap 2 weeks 4 weeks |
| Step 11 |  |
| Scoring experimental probes | Alongside sessions?  And tidy up at end of |

## Step 1: Teacher briefing and set up.

1. Briefing: Teacher and school briefed that the focus of the study is not EAL but bilingual children with language disorder. If home language is well-developed but English is not, then the child is NOT suitable for intervention. If the child has problems in home language with sufficient exposure then they WOULD be suitable. Discuss languages targeted by study - Mirpuri, Sylheti or Polish.

## Step 2: Teacher identifies candidate children.

1. Identification: Teacher asked to approach families of children who
   - *Speak Mirpuri, Sylheti or Polish at home, teachers are asked to pose question to parents as a choice – e.g. ‘do you speak Punjabi, Mirpuri or Urdu?’; ‘do you speak standard Bangla or Sylheti?’*
   - Aged between 3; 02 and 4; 05 years
   - Can engage in pretend play and shares attention with adults
   - Are not making expected progress in English after entry to Nursery compared to peers from same heritage backgrounds
   - Have no associated diagnosis
     - Sensorineural hearing impairment
     - Severe visual impairment
     - Diagnosed learning disability
2. Preparation: Teacher asked to ask parents what the child’s language at home is like – answers such ‘s/he talks very little’ would suggest worth following up.
3. Teacher to discuss types of sentences child uses at home in home language with parents and note information when passing on details to research team What kinds of sentences does your child usually say? Can you give me an example?
4. Teacher to discuss case with EAL services if possible and determine whether they would judge the child as having poorer home language than they would expect given the exposure and age of the child

INSERT LINKS TO RELEVANT RESOURCES

## Step 3: Consent obtained from family for Assessment & Study

1. Meet and brief interpreter and talk through process. Record video of information sheet.
2. Teachers give parents information sheets in English but with visual instruction as to how to access video recordings of interpreters talking through information sheet in relevant language.
3. Invited to meeting in school with RA and interpreter to talk though in more detail
4. Interpreter confirms languages spoken in the home
5. Meet in school to talk through information sheet and consent form with interpreter present - Interpreter countersigns the consent form to state that they believe the family have understood all the information presented and consent.

INSERT LINKS TO RELEVANT RESOURCES

## Step 4: Assessment

Preparation - Hold preparatory training meeting with interpreter to talk through their role in supporting assessment administration

Essential Assessments

1. Language Exposure and Language Development ‘Questionnaire’: Used as semi-structured interview with parent/caregiver conducted by therapist with support from interpreter, this will be for background information but not part of the assessment criteria
2. Teacher Questionnaire: Teacher completes modified section 6 of the questionnaire about Home Language and English to identify their concerns
3. FOCUS: Parent competes FOCUS with interpreter – use a visual aid to support use of Likert scale
4. FOCUS: Teacher completes FOCUS with therapist
5. Play Activity: Assessor uses Home Language with some English Language code switching to encourage/make code switching ‘allowable’. Therapist notes BEST eligibility check criteria (pretend play – triadic attention)
6. Home Language BEST Assessment- – Code-switching credited
7. Home Language NRDLS Comprehension Sections A – D – Code-switching credited
8. Home Language NRDLS Production Sections A – D – Code-switching credited
9. BEST assessment in English

Suggested structure

- Set up meeting – FOCUS & Language use/development questionnaire
- Session 1. Play Activity and Home Language Assessments
- Session 2. BEST in English and discussion with family and school

INSERT LINKS TO RELEVANT RESOURCES

## Step 5: Comparison to Eligibility Criteria

Key principle for eligibility: child would benefit from BEST – i.e. to reach age expected levels in home language they need to improve in one or more of the following

- Develop use of 3 and 4 clause element sentences
- increase free and bound grammatical morpheme use
- increase range of verbs they use in sentences
- Increase correct usage of key inflections (see below), where present in the language

Essential Criteria

1. Home Language BEST assessment: identifies difficulties in any of above aspects (Arg Structure – morphology – agreement – verb knowledge/use) allowing for code-switching to be accepted.
2. A score of ≤ 75% score on the PAS score or – where applicable on a scoring system allowing for missing agent such that child is credited with the agent if they omit it but use the correct 3ps verb form.

AND

3. NRDLS assessment: comprehension score and/or NRDLS production score 25/40 or below plus less than 5/10 on either sections C or section D (or both) allowing for code-switching to be accepted

**Things to note:**

**Mirpuri**

Signals the Subject gender via the tense marker on the verb phrase (either directly onto the lexical verb, and/or a ‘dummy do’ for the composite light verbs, and on the auxiliary for Mirpuri). Nazir (2014) agrees and describes such simple sentences as ‘kher-na eh” as an empty set S(O)V, that is Ø / lexical verb + male (natural gender of the person playing) + present tense + auxiliary) S V.

**Sylheti**

Does **not** encode natural gender and the verb phrases are the same regardless of the gender of the Subject (AGENT), so, unlikely to be pro-drop.

**Polish**

All nouns potentially have a case ending, although for some (mainly nominative) this is ‘zero’. Case inflections therefore play a role in thematic role assignment. Word order is free, but there appears to be a preference for SVO (canonical word order?). There are several ‘conjugations’ for verbs and person/number involves changes to the whole word rather than addition of a particular inflection. It is a pro-drop language, so selection of correct verb form can replace subject pronoun use.

There are no determiners in Polish.

Supplementary Criteria

***If the child has a ‘comprehension only’ profile*** they are not eligible for the study

***If the child provides only limited response*** ***in home language*** analyse BEST in English and consider NRDLS in English

***If the child is a borderline/unclear case on Essential Criteria*** consider 1) code-switching and 2) FOCUS

1. Code switching of nouns and verbs (verbs which have home language verb structure intact) is a positive sign and would indicate non-eligibility. Missing code-switching would sway a borderline case towards indicate eligibility.
2. FOCUS – score indicative of significant activity and participation limitations – [Score of less than 4 on > 80% of the 34 items (ie ~ 28 items)

Note: by using scoring allowing for code-switching and creating context which might encourage code-switching we should be able to determine if difficulties exist in ‘sequential bilingual’/ child with more balanced exposure to languages i.e. if not impaired should be able to use code-switching to reach near age-appropriate levels of argument structure use and apply morphological frame.

## Step 6 Intervention delivery set up

1. Let parents know about outcome of above assessment and explain intervention if eligible

2. Train interpreter and plan delivery

## Step 7: Randomisation to baseline set

- Children randomised to different baseline period – ask non-project member to draw number from ‘hat’ to identify baseline A, B or C – want variety so will draw from set of 3 until all 3 (ABC) used then add ABC to hat again for 4^th^ (if do more than 3).
- All children also receive baseline period where complete picture description assessment 2-3x per week for either (A) 5 sessions (over 2 weeks), (B) 8 sessions (over 3 weeks) or (C) 11 sessions (over 5 weeks) (total of 10 weeks or 11 weeks or 12 weeks)
- All children have 16 intervention sessions delivered over 8 weeks

## Step 8: Baseline assessments

- For the baseline period children will describe a set of 30 pictures each session made up of a random selection of BEST homework pictures (N = 114) – child chooses reward game from – posting with burping gorilla – popup pirate – etc (set of 6 games? And can change halfway if bored).
- All sessions videoed
- Transcription and Scoring completed after session by administrator of assessments with therapist support
- Sample of 10% of these videos and assessments completed during therapy transcribed and scores by researcher blind to week of therapy

## Step 9: Intervention delivery & Assessments

- Delivery of 16 sessions of BEST over 8 weeks in home language by interpreter with SLT support
- At each session also complete picture description assessment (as above – 30 pictures random selection and video)
- At Session 5 Video BEST session and complete treatment fidelity check

## Step 10: Outcome Assessment

Complete over 3 sessions

- BEST Assessment
- NRDLS
- FOCUS
- at each session also complete 30 picture probe.
- Semi-structured interview with partners (parents/interpreter/school staff) re implementation barriers and enablers

## Step 11: Scoring experimental probes

- All sessions videoed
- Transcription and Scoring completed after session by administrator of assessments
- Sample of 10% of baseline videos and assessments completed during therapy transcribed and scored by researcher blind to week of therapy

Key additional considerations

## Interpreters

- Need to add up the total number of hours per child including training and briefing
- Need to then get quote for costs

## RA resource

- RA has 2.5 days per week almost entirely for LIVELY next year Work with 3 children, or 4 - if resources available – interpreters and identified children

# Language Exposure and Development Questionnaire

**We would like to know about who your child talks to and what languages he or she speaks. Please answer the following questions**

1. ***LIVING IN THE UK***

Was your child born in the UK?

**Answer yes or no:**

If no, how old was your child when they came to the UK?

**Age …..years…..months**

2. ***AT HOME***

What languages do you speak at home?

**Languages:**

Does your child have any brothers or sisters?

**Answer yes or no:**

**If yes, how old are they, and what language or languages does your child speak when with them?**

| Child | Age (years) | Languages used |
| --- | --- | --- |
| Brother/Sister 1 |  |  |
| Brother/Sister 2 |  |  |
| Brother/Sister 3 |  |  |
| Brother/Sister 4 |  |  |
| Brother/Sister 5 |  |  |
| Brother/Sister 6 |  |  |

When your child is at home, what language or languages does he or she speak for everyday family activities (e.g. mealtimes, getting ready for bed).

**Language(s):**

Does your child do activities with other people living in the home who are not his/her parents or brothers or sisters (e.g. grandparents)?

**Answer yes or no:**

If yes what language(s) do they use?

**Language(s):**

Does your child spend time with visitors to the home?

**Answer yes or no:**

If yes, what language(s) do they use?

**Language(s):**

Does your child watch television/videos or play computer games at home?

**Answer yes or no:**

If yes, how much time do they spend doing this each week, and what languages do they hear?

**Time (hours):**

**Language(s):**

3. ***OUTSIDE HOME***

Does your child talk with relatives who don’t live near you through e.g. Skype or telephone?

**Answer yes or no:**

**If yes, how often (approximately) and in which language(s)?**

| **Frequency** | **Language(s)** |
| --- | --- |
| Once per day or more |  |
| 3 or more days a week |  |
| Once a week |  |
| Once a month |  |
| Less than once a month |  |

When did your child start coming to XXX school/nursery?

**Date:**

Other than school, what other things does your child do outside the home (e.g. visits to friends/relatives, visits to the park, religious activity, festivals and other celebrations, etc.)? What language do they speak for these?

| **Activity** | **Language(s)** |
| --- | --- |
|  |  |
|  |  |
|  |  |
|  |  |
|  |  |

4. ***WHEN YOUR CHILD WAS YOUNGER***

Before your child started coming to school, who looked after him/her? What languages do they speak:

**You………..**

**Language…..**

**Other Family members……..**

**Language…**

**Non family members (e.g. friends, child minder)…**

**Language……**

Page Break

5. ***TIME AWAY***

Has your child spent any time outside the UK (for periods longer than a week) in the last 6 months?

**Answer yes or no:**

If yes, tell us where they went and what languages did they hear.

**Where?......**

**Language(s) spoken:**

# Home Language Development - Parent

In these questions we are interested in how your child uses your **home language** – **[Sylheti/Polish/Mirpuri]**.

1. How do you feel your child’s use of home language compares to other children of their age?
2. Are you worried that your child’s talking is behind or slower than you expect?
3. When you ask your child to do something for you, like go and get their shoes and coat, can they understand you?
4. When your child wants something or wants to tell you something, what do they do to let you know?
5. If you asked your child a question about something that happened yesterday could they tell you clearly? For example, if you asked them what they did at nursery?
6. How easy do you find it to understand what your child says to you? Would you say you understand your child when they speak to you = all of the time – most of the time – some of the time – rarely – never
7. Now we want to think about people who don’t know your child well. Do you ever have to explain to others what your child has said?
8. How often do other people understand what your child says when they speak to them - all of the time – most of the time – some of the time – rarely – never
9. It can help us to get an idea of your child’s language development if we can think of three examples of their longest sentences you have heard them say.

- What would you say is the longest sentence you have heard?
- Can you think of another example?
- And another?

# Language Development – Teacher

In these first questions we are interested in how [child’s name] uses **English in nursery**

1. How do you feel [child’s name] use of English compares to other children of their age, language background and who have spent a similar time in nursery?
2. Are you worried that their talking is behind or slower than you expect?
3. When you ask [child’s name] to do something for you in English, like go and get their shoes and coat, can they understand you?
4. If you asked [child’s name] a question about something that happened yesterday in English could they tell you clearly? For example, if you asked them what they did at nursery?
5. How easy do you find it to understand what [child’s name] says to you? Would you say you understand them when they speak to you = all of the time – most of the time – some of the time – rarely – never
6. Now we want to think about people who don’t know [child’s name] well. Do you ever have to explain to others what [child’s name] has said?
7. How often do other people understand what [child’s name] says when they speak to them - all of the time – most of the time – some of the time – rarely – never
8. It can help us to get an idea of [child’s name] language development if we can think of three examples of their longest sentences you have heard them say.

- What would you say is the longest English sentence you have heard them say?
- Can you think of another example?
- And another?

In these next questions we are interested in how [child’s name] uses **their home language – [Sylheti/Polish/Mirpuri]**.

You may not have all the answers but if you can answer to the best of your knowledge drawing on information from discussions you have had with [child’s name] parents and EAL teacher or observations of them speaking to staff who also speak their home language.

9. How do you feel [child’s name] use of [home language] compares to other children of their age and language background?
10. Are you worried that their talking in their home language is behind or slower than you expect?
11. When people ask [child’s name] to do something for them in [home language], like go and get their shoes and coat, can they understand them?
12. If people ask [child’s name] a question about something that happened yesterday in [home language] could they tell them clearly? For example, if they asked them what they did at nursery?
13. How easy do people who speak the child’s home language find it to understand what [child’s name] says? Would they say they understand them when they speak to them = all of the time – most of the time – some of the time – rarely – never
14. It can help us to get an idea of [child’s name] language development if we can think of three examples of their longest sentences you have heard them say in [home language]. Do you feel you or a member of your nursery staff would be able to comment on that?

- What would you say is the longest [home language] sentence you have heard them say?
- Can you think of another example?
- And another?

# Appendix

**Changes to assessments/interventions**

1. **NRDLS**: issue with ‘waving’

**Comprehension section Ci:** child is asked to make Monkey perform actions. Currently *jump, run* are practice items, and *sit, walk, clap, wave, hide* are the test items. I suggest we replace the test item *waving* with *running*, and use *pointing* as a practice item. It is not possible to make Monkey perform a precise pointing action (as he has no fingers) but this should work OK as a practice item.

So, the section would be then be:

Practice - 'make Monkey...*jump, point'*

Test items - 'make Monkey...*sit, walk, clap, run, hide.*

**Comprehension section cii:** picture selection from a choice of four. Practice items are 'show me Monkey ..*sitting, waving*. Test items - *reading, sliding, flying, blowing, pointing.* I suggest we substitute *walking* for *waving* in the practice items, which is another picture in the same set. Then no further change needed to test items.

For **Production, section Ci:** tester makes Monkey carry out action and asks child what Monkey is doing. Currently Practice items are *jump, run* and test items are *wave, clap, walk, sit, fall*. I suggest we substitute *stand* for *run* in the practice items, with tester moving Monkey from sitting to standing position, and then substitute *run* for *wave* in the test items.

Section then becomes:

Practice - *jump, stand*

Test items - *run, clap, walk, sit, fall.*

**Production Section Cii**: tester shows child a picture and asks 'what is Monkey doing?' Practice items are: *sit, wave.* Test items are: *swim, slide, fly, read. point*.

I suggest we just drop *wave* from the practice items and just have one practice item. This would avoid having to make new picture.

2. **BEST intervention (Polish)** issue with ‘ride’

The main issue is the verb *ride* which in English can be a transitive verb with an object, but in Polish seems to only work as verb followed by a prepositional phrase (*ride on a horse, etc*).

CM: I have decided we should put brush in place of ride for set D in Polish - brush is currently only used in the output phase - I realise this makes it different to all the other verbs in that it is in there more frequently and in input and output but given we are not comparing the different verbs I think this is OK - my rationale is 1) the agents and patients are both animate in riding and brushing; 2) the patients are similar in riding and brushing; 3) brushing has fewer exposures than many of the other verbs in terms of the items per session.

Updated intervention to replace *ride* with *brush* – ‘ride a bike’ > now ‘brush a teddy’
